# Supplementary figures and images for: Reducing target binding affinity improves the therapeutic index of anti-MET antibody-drug conjugate in tumor bearing animals
Source: PLoS One. 2024 Apr 17;19(4):e0293703. doi: 10.1371/journal.pone.0293703 (PMC11023234; doi:10.1371/journal.pone.0293703)

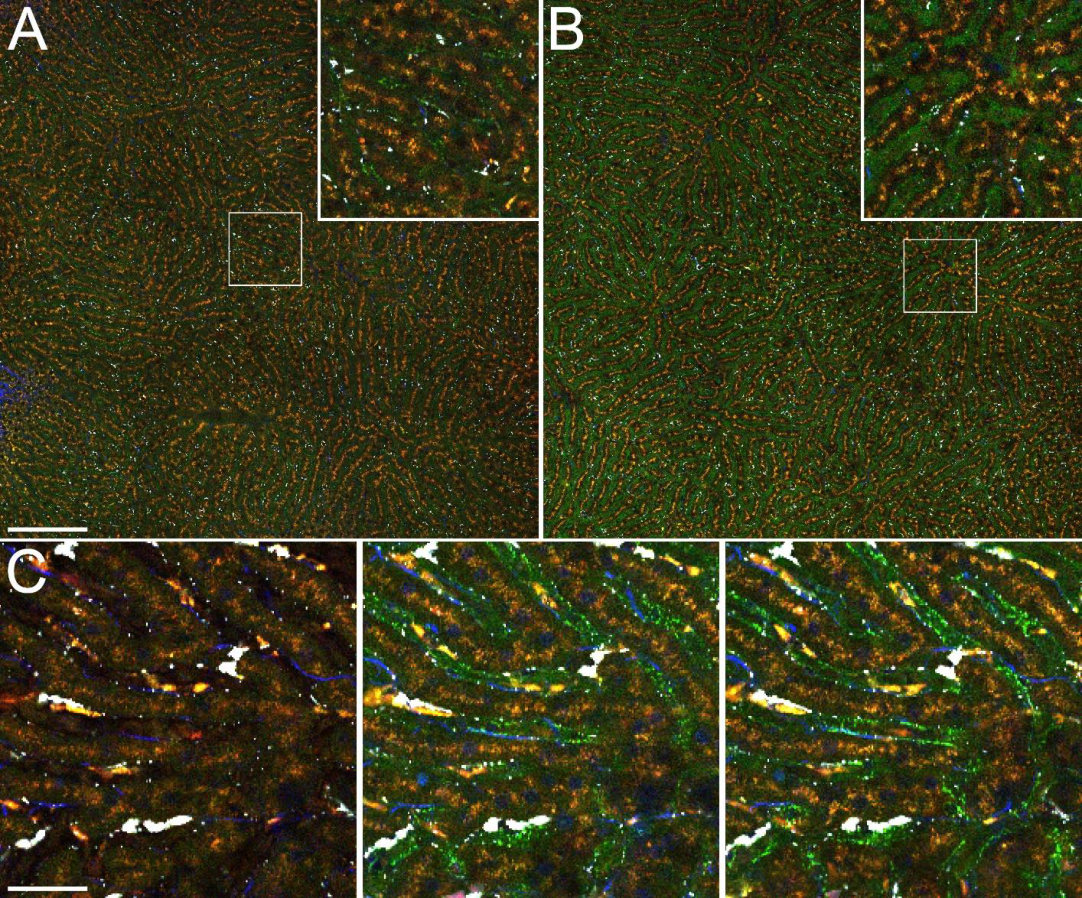

Supplement: S1 Fig — Multiphoton fluorescence excitation images of the livers of living Munich-Wistar-Fromter rats were collected 2 hours after intravenous injection of 6 mg/kg DL488-HAV (Panel A) or DL488-LAV (Panel B). Indicated regions are reproduced at 3X magnification in insets. Similar to the results obtained at 24 hours, punctate fluorescence is observed in rats injected with DL488-HAV but not DL488-LAV. (C) Time series of images collected from the same region of the liver prior to (left panel) and then again 1 and 2 hours after intravenous injection of DL488-HAV (middle and right panels, respectively). In this study, DL488-HAV accumulation was particularly pronounced, becoming apparent within one hour, and continuing to accumulate over the next hour. Neither construct is detectibly associated with hepatocytes, as identified by their brown autofluorescence, arranged in linear “cords”. Bright white punctate fluorescence reflects broad-spectrum autofluorescence of vitamin A in stellate cells. Blue signal derives from second harmonic generation, likely from collagen. Scale bars represent 200 microns in panels A and B, and 50 microns in panel C. All images are collected using identical microscope settings and contrast enhanced identically. (TIF) [file pone.0293703.s001.tif]

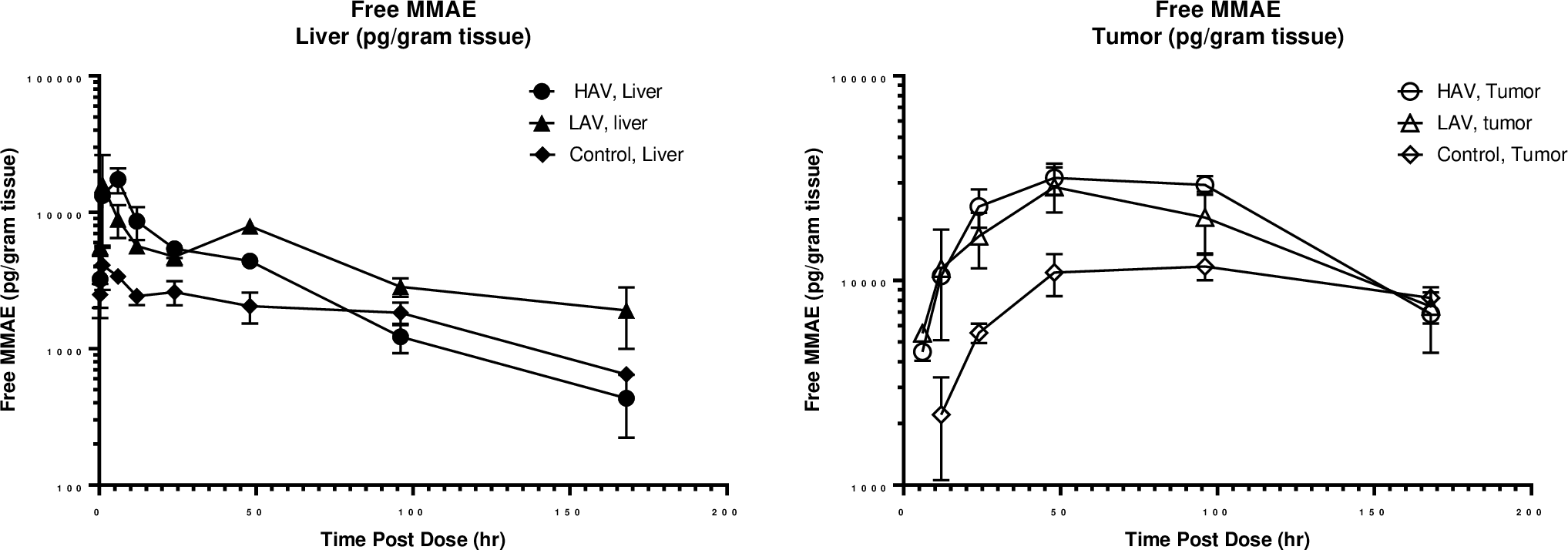

Supplement: S2 Fig — Pharmacokinetic profiles of free MMAE (payload) following a single 6 mg/kg IV administration of the isotype control ADC or LAV and HAV ADCs within liver and tumor tissues in HT29 tumor bearing rats. Data are the mean (+/-SD) concentrations for three animals/timepoint for each of the molecules. The free MMAE exposure following administration of the ADCs were characterized for 168 hours post the single administration. Non-serial sample with N = 3 rats/time point was leveraged as tumor and liver collections involved terminal sample collection. (TIF) [file pone.0293703.s002.tif]
